# Genetic *trans*-Complementation Establishes a New Model for Influenza Virus RNA Transcription and Replication

**DOI:** 10.1371/journal.ppat.1000462

**Published:** 2009-05-29

**Authors:** Núria Jorba, Rocío Coloma, Juan Ortín

**Affiliations:** Centro Nacional de Biotecnología (CSIC) and CIBER de Enfermedades Respiratorias, Campus de Cantoblanco, Madrid, Spain; Harvard Medical School, United States of America

## Abstract

The influenza A viruses genome comprises eight single-stranded RNA segments of negative polarity. Each one is included in a ribonucleoprotein particle (vRNP) containing the polymerase complex and a number of nucleoprotein (NP) monomers. Viral RNA replication proceeds by formation of a complementary RNP of positive polarity (cRNP) that serves as intermediate to generate many progeny vRNPs. Transcription initiation takes place by a cap-snatching mechanism whereby the polymerase steals a cellular capped oligonucleotide and uses it as primer to copy the vRNP template. Transcription termination occurs prematurely at the polyadenylation signal, which the polymerase copies repeatedly to generate a 3′-terminal polyA. Here we studied the mechanisms of the viral RNA replication and transcription. We used efficient systems for recombinant RNP transcription/replication *in vivo* and well-defined polymerase mutants deficient in either RNA replication or transcription to address the roles of the polymerase complex present in the template RNP and newly synthesised polymerase complexes during replication and transcription. The results of *trans*-complementation experiments showed that soluble polymerase complexes can synthesise progeny RNA *in trans* and become incorporated into progeny vRNPs, but only transcription *in cis* could be detected. These results are compatible with a new model for virus RNA replication, whereby a template RNP would be replicated *in trans* by a soluble polymerase complex and a polymerase complex distinct from the replicative enzyme would direct the encapsidation of progeny vRNA. In contrast, transcription of the vRNP would occur *in cis* and the resident polymerase complex would be responsible for mRNA synthesis and polyadenylation.

## Author Summary

The influenza A viruses produce annual epidemics and occasional pandemics of respiratory disease. There is great concern about a potential new pandemic being caused by presently circulating avian influenza viruses, and hence increasing interest in understanding how the virus replicates its genome. This comprises eight molecules of RNA, each one bound to a polymerase complex and encapsidated by multiple copies of the nucleoprotein, in the form of ribonucleoprotein complexes (RNPs). These structures are responsible for virus RNA replication and transcription but the detailed mechanisms of these processes are not fully understood. We report here the results of genetic complementation experiments using proficient *in vitro* and *in vivo* recombinant systems for transcription and replication, and polymerase point mutants that are either transcription-defective or replication-defective. These results are compatible with a new model for virus replication whereby a polymerase distinct from that present in the parental RNP is responsible for RNA replication *in trans* and the progeny RNP is associated to a polymerase distinct from that performing replication. In contrast, transcription is carried out *in cis* by the polymerase resident in the RNP.

## Introduction

The influenza A viruses are the causative agents of yearly epidemics of respiratory disease and occasionally more severe pandemics [1]. The latter are the consequence of transfers from the avian virus reservoir to humans by either genetic reassortment or direct adaptation [2]. Thus, current occasional infections of humans with highly pathogenic H5N1 avian strains have raised fears about a possible new pandemic of great severity.

The influenza A viruses belong to the family *Orthomyxoviridae* and posses a single-stranded, negative-polarity RNA genome made up by 8 RNA segments, that form ribonucleoprotein (RNP) complexes by association to the polymerase and the nucleoprotein (NP). Such RNPs are independent molecular machines responsible for transcription and replication of each virus gene and contain an RNA-dependent RNA polymerase composed by the PB1, PB2 and PA subunits [3]. The polymerase complex recognises the RNA promoter comprising both 5′-terminal and 3′-terminal sequences of each segment, by preferentially binding the 5′-terminal end [4]–[6], and in this way stabilises a supercoiled conformation of the RNPs [7].

Upon infection of susceptible cells, the parental RNPs are first transcribed in the nucleus (primary transcription). Transcription initiation takes place by a cap-snatching process whereby the viral polymerase recognises the cap structure of cellular pre-mRNAs in the nucleus, cleaves these some 15 nt downstream the cap and utilises such capped-oligonucleotides as primers to copy the virus template RNA [8]. Transcription finalises by reiterative copy of the virus polyadenylation signal, an oligo-U sequence located close to the 5′-end of the template [9],[10]. Synthesis of new virus proteins is required to proceed to RNP replication [11], that takes place first by the generation of complementary RNPs (cRNPs). These RNPs are structurally analogous to those present in the virions (vRNPs) but contain complete positive-polarity copies of the virus RNA segments, that are neither capped nor polyadenylated. The structural differences between the vRNP transcription and replication products (mRNAs and cRNPs) led to the proposal of a transcription-to-replication switch by which the parental RNPs would change from capped-RNA-dependent to *de novo* initiation, from polyadenylation to full copy of the template, and in addition would induce encapsidation of the RNA product into new RNPs (reviewed in [12]. Such notion has been challenged recently by a new model proposing that parental vRNPs can directly synthesise cRNA but require newly synthesised polymerase and NP to stabilise the product in the form of cRNPs [13]. The cRNPs accumulate to low levels but serve as efficient templates for the synthesis of large quantities of progeny vRNPs that can be transcribed (secondary transcription) and eventually be incorporated into progeny virions [3].

Much information has been obtained during recent years on structural aspects of the RNPs [14],[15] (R. Coloma, unpublished results) and their components, like the NP [16],[17], the polymerase complex [18],[19] and specific domains of the polymerase subunits [20]–[24]. Likewise, a number of host cell factors have been identified that may play important roles in the transcription and replication processes [25]–[34]. However, much remains to be learned about the detailed mechanisms for RNP transcription and replication. For instance, it is not clear whether the polymerase complex present in the template RNP is able to synthesise the progeny vRNA or whether the replicative complex directs the encapsidation of progeny RNA it into a new vRNP. Likewise, it has been assumed that the polymerase complex present in the vRNP accounts for viral mRNA synthesis, but it is not clear whether other vRNPs or other soluble polymerase complexes perform this step *in trans*. In this report we used efficient in vivo recombinant replication and transcription systems and defined polymerase mutants specifically affected in either transcription or replication to answer these questions. Our results are consistent with a new model whereby polymerase complexes not associated to the template cRNP are responsible for the replicative synthesis of vRNPs *in trans* and polymerase complexes distinct from the replicative one specify the encapsidation of viral RNAs. On the contrary, no vRNP transcription could be detected by other RNP or a soluble polymerase complex *in trans*, suggesting that it takes place by the activity of the RNP-associated polymerase complex.

## Results

### The experimental approach

To gather information on the mechanisms of influenza virus transcription and replication we have adopted a genetic *trans*-complementation approach. This is based on the ability to reconstitute in vivo an efficient transcription-replication system that mimic these steps of the infection cycle and is more amenable to experimental manipulations [15],[35]. Furthermore, the vRNP products can be efficiently purified, their structural and biological properties can be easily analysed [14],[18],[20] and they can in turn be used as templates for further rounds of in vivo replication. Essential for these approaches is the availability of well-defined mutants to be used as genetic markers. We have earlier described point mutants in the PB2 subunit of the viral polymerase that are defective in viral RNA replication but fully efficient in virus transcription [36]. Likewise, we have recently reported polymerase PB2 mutants that are affected in the cap-binding activity and hence are defective in cap-snatching, but retain their capacity to replicate virus RNPs [20].

### A polymerase complex distinct from the replicative enzyme becomes associated *in trans* to the newly synthesised vRNA

**Figure 1:**
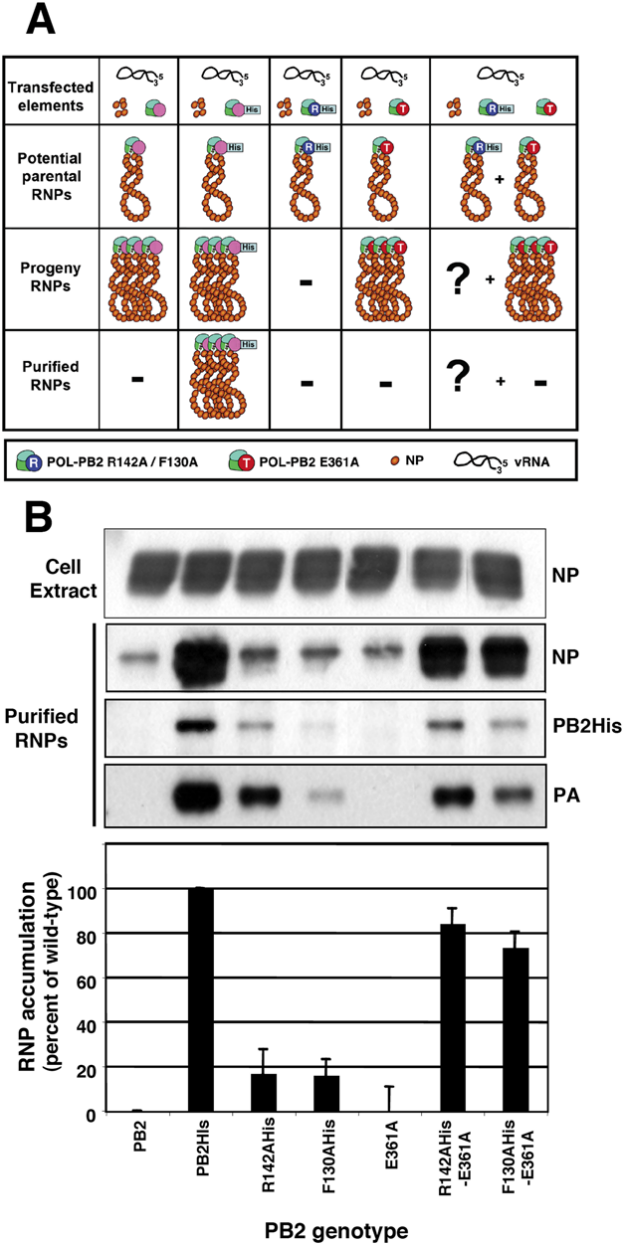
Intracistronic polymerase complementation during influenza virus RNA replication. (A) Cultures of HEK293T cells were transfected with plasmids expressing a virus-like replicon of 248 nt, the NP and various combinations of the polymerase subunits as indicated in the diagram. The potential RNPs that could be generated are also depicted in the diagram, as well as the expected progeny RNPs, depending on the replication phenotype of the polymerase mutants used. (B) The progeny RNPs were purified from total cell extracts over Ni^2+^-NTA-agarose resin and analysed by Western-blot with anti-NP antibodies. The top panel presents the accumulation of NP in the total cell extract whereas the bottom panel shows the NP accumulation of purified RNPs. The integrity of the purified RNPs is verified by Western-blot using anti-PB2 and anti-PA antibodies. In the bottom graph the average NP accumulation and standard deviation of three independent complementation experiments are presented as percent of maximal value.

Using the approaches indicated above we first addressed the question whether the replication deficiency of point mutants within the N-terminus of PB2 [36] could be rescued *in trans* by co-expression of PB2 point mutants defective in cap-binding [20]. Cultures of HEK293T cells were co-transfected with plasmids encoding PB1, PA, NP and a deleted NS virus replicon (clone 23, 248 nt in length; [14],[15]). In addition, either PB2wt or PB2 mutants R142A or F130A (replication-defective) or mutant E361A (transcription-defective) were co-expressed. Alternatively, pair wise combinations of these PB2 mutants were co-expressed (R142A+E361A and F130A+E361A). Among the PB2 proteins expressed, either wt or the replication-defective mutants R142A or F130A were His-tagged at the C-terminus, a modification that does not alter their biological activity and allows the efficient purification of the in vivo RNP replication progeny [18]. The expression levels of all PB2 mutants were shown to be similar to that of PB2wt (Fig. S1) and the untagged PB2wt was used as a control for purification (see diagram of the experimental setting in Fig. 1A). After incubation, the cell extracts were used for Ni^2+^-NTA-agarose purification as described in Materials and Methods and the accumulation of progeny RNPs was determined by means of Western-blot assays using anti-NP sera. The purification of the complete RNPs was verified by Western-blot with antibodies specific for PB2 and PA (Fig. 1B). This strategy allows measuring the replication capacity of the RNPs formed in vivo, as omitting any RNP element or using a defective point mutant leads to undetectable RNP accumulation [15],[36],[37]. Amplification of virus RNPs was expected for wt and mutant polymerase containing transcription-defective PB2 (E361A), but not for those containing replication-defective PB2 (R142A and F130A). However, since no tag is present in the former mutant, only RNPs derived from cultures containing PB2His were expected in the Ni^2+^-NTA-agarose purified material. This was indeed the case, as shown in Fig. 1B. If the transcription-defective polymerase were able to rescue *in trans* the defect in replication of polymerase mutants R142A or F130A, one would expect the accumulation and purification of RNPs containing these mutant PB2. The results obtained by the co-expression of pairs of replication- and transcription-defective polymerases indicate that such prediction is hold (Fig. 1B). The transcription-defective mutant could rescue both R142A and F130A alleles and similar rescue was obtained when other transcription-defective mutants, like H357A, K370A, F404A [20] were used (Fig. S2).

**Figure 2:**
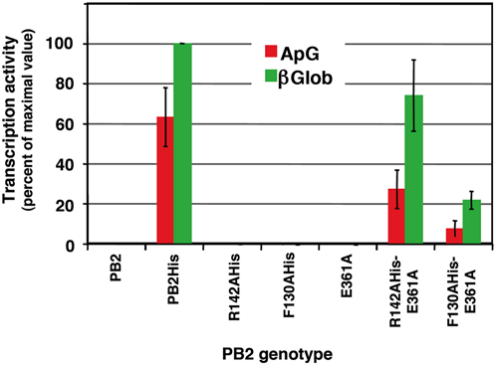
Phenotype of *trans*-complemented RNPs. The purified RNP preparations presented in Fig. 1 were tested for in vitro transcription primed with either ApG (red) or β-globin mRNA (green). The data are presented as percent of maximal value and represent the averages and ranges of two independent complementation experiments. The transcription activities parallel the values of NP accumulation presented in Fig. 1 and show that the rescued RNPs have a wt cap-snatching phenotype.

The progeny RNPs contained the replication-defective PB2 allele, since (i) they could be purified by Ni^2+^-NTA-agarose chromatography and (ii) the mobility of the PB2 subunit in the Western-blot assay corresponded to the His-tagged subunit and not to the untagged one. It is important to mention that only His-tagged PB2 protein was detected in the purified RNPs and not the untagged counterpart, indicating that no transcription-defective polymerase was co-purified (Fig. 1B). Furthermore, the phenotype of the rescued RNPs was tested by determination of their in vitro transcription activity (Fig. 2). Since the transcription-defective mutants had alterations in their cap-binding pocket, they show low in vitro transcription activity when a mRNA is used as a cap-donor, whereas cap-independent transcription is observed with a general primer as the dinucleotide ApG [20]. The transcription activity profile of rescued RNPs using ApG or β-globin mRNA as primers was identical to that of wt RNPs, as expected, and not to that of mutant E361A, that is unable to use β-globin as primer [20] (Fig. 2 and Fig. S3).

In the experimental approach used, the reconstitution of a RNP from the viral proteins and genomic RNA has to take place first and its subsequent amplification would account for the bulk of RNPs that can be purified from the transfected cells. Since a RNP template with the replication-defective polymerase does not replicate [36], only transcription-deficient polymerase could perform RNP replication. The results obtained (Figs. 1, 2) demonstrate that a polymerase complex distinct from that responsible for RNP replication (replication-defective versus transcription-defective) is incorporated into the progeny vRNP and suggest that a replication-defective polymerase can direct the encapsidation of the progeny vRNA, i.e. can bind the 5′-terminus of newly synthesised vRNA and direct the incorporation of NP monomers into the progeny vRNP. It could be argued that the incorporation of the replication-defective polymerase to the progeny RNP might occur by exchange with replication-competent during purification in vitro. Two lines of evidence argue against such possibility: (i) Our transcription experiments verify that the polymerase present in an RNP complex is stably bound (see below) and (ii) The data reported by Wreede et al. [38] suggest that the binding of a polymerase complex to the 5′-terminal sequence of viral RNA can not competed by a pre-expressed polymerase. In fact, the average rescue efficiency obtained (55+/−18%) (Figs. 1, 2) was very high, and is in line with the possibility that both types of soluble polymerase complexes, transcription- and replication-deficient, are incorporated in the progeny viral RNA, around half of which would not be detected because are not His-tagged.

### Non-resident polymerase complexes are responsible for the synthesis of vRNA *in trans*

**Figure 3:**
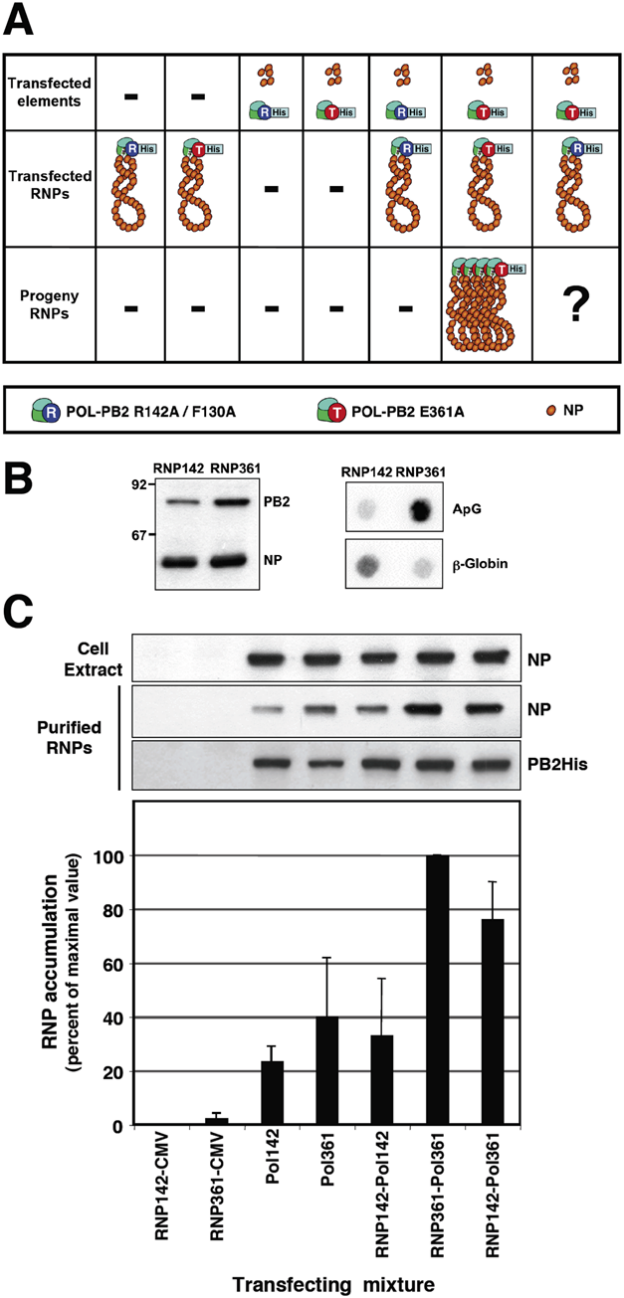
Replication of RNPs by a soluble polymerase complex *in trans*. (A) Cultures of HEK293T cells were transfected with plasmids expressing the NP and various combinations of the polymerase subunits as indicated in the diagram. In some cases, purified RNPs containing replication-deficient or transcription-deficient polymerase were also transfected. The expected progeny RNPs are also depicted, depending on the replication phenotype of the polymerase mutants used. (B) Left panel: The amount of replication-deficient (R142) or transcription-deficient (R361) RNPs transfected was ascertained by Western-blot with anti-NP and anti-PB2 antibodies. The mobility of molecular weight markers is shown to the left and the position of PB2 and NP proteins is indicated to the right. Right panel: The transcription phenotype of the RNPs transfected was determined by in vitro transcription using ApG (ApG) or β-globin mRNA (β-glob) as primers. The panel shows the phosphorimager data. (C) The progeny RNPs were purified from total cell extracts over Ni^2+^-NTA-agarose resin and analysed by Western-blot with anti-NP antibodies. The top panel presents the accumulation of NP in the total cell extract whereas the bottom panel shows the NP accumulation of purified RNPs. The integrity of the RNPs is verified by Western-blot using anti-PB2 and anti-PA antibodies. In the bottom graph the average NP accumulation and range of two independent complementation experiments are presented as percent of maximal value. The transfecting RNPs are denoted as RNP142 or RNP361. The genotypes of the transfected polymerases are indicated as Pol142 or Pol361. CMV indicates the transfection of empty pCMV plasmid.

**Figure 4:**
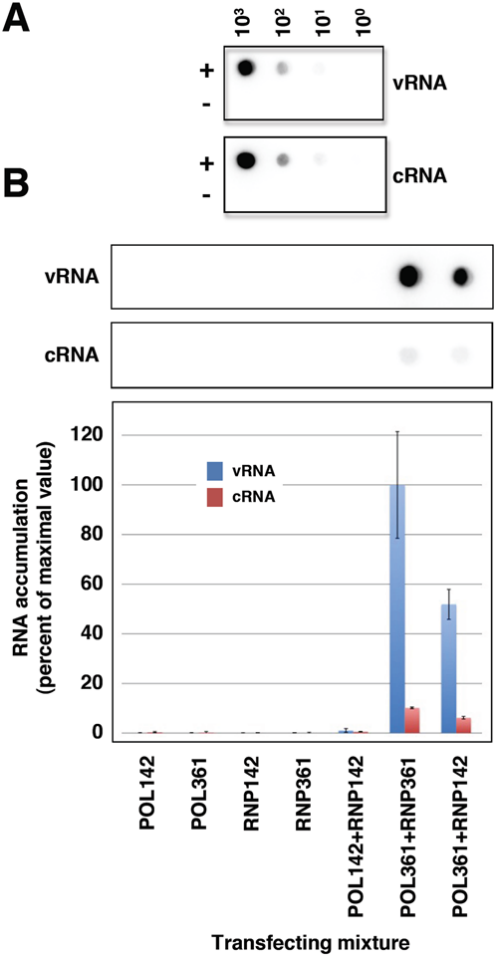
Analysis of the genomic RNA present in purified RNPs. Cultures of HEK293T cells were transfected with plasmids expressing the NP and various combinations of the polymerase subunits and purified RNPs containing either replication-deficient or transcription-deficient polymerase, as indicated in the diagram of Fig. 3A. The purified progeny RNPs were purified from total cell extracts by affinity chromatography over Ni^2+^-NTA-agarose and the RNA present in the purified RNPs was extracted as described under Materials and Methods. (A) Hybridisation controls. Dilutions of plasmid pHHΔNS clone 23, containing the sequence of the RNP replicons used (+), or total yeast RNA (−) were applied onto a nylon filter as hybridisation controls (from 10^3^ to 10^0^ ng, as indicated at the top of the figure). Hybridisation was performed using a positive-polarity or a negative-polarity probe comprising the full-length insert present in pHHΔNS clone 23, thereby detecting either vRNA or cRNA, respectively. (B) Aliquots of the RNA present in purified RNPs obtained from cultures transfected with the mixtures indicated at the bottom of the figure were hybridised in parallel to the hybridisation controls shown in (A) and the hybridisation signals were quantitated in a phosphorimager, using the signals in (A) to standardise the relative hybridisation efficiency of the positive- and negative-polarity probes. The results for vRNA (blue) and cRNA (orange) are presented as percent of the maximal signal and represent the averages and standard deviations of 4 quantisations.

**Figure 5:**
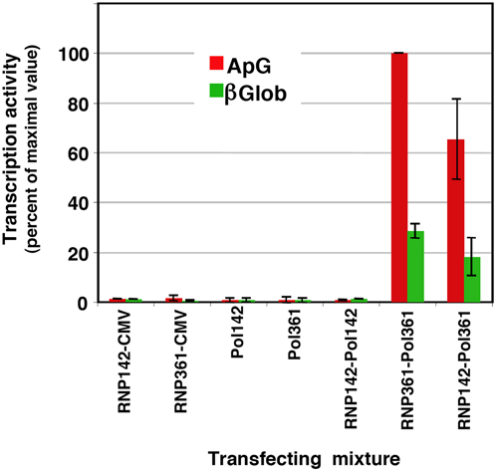
Phenotype of *trans*-complemented RNPs. The purified RNP preparations presented in Fig. 3 were tested for in vitro transcription primed with either ApG (red) or β-globin mRNA (green). The data are presented as percent of maximal value and represent the averages and range of two independent complementation experiments. The transfecting RNPs are denoted as RNP142 or RNP361. The genotypes of the transfected polymerases are indicated as Pol142 or Pol361. CMV indicates the transfection of empty pCMV plasmid. The transcription activities parallel the values of NP accumulation presented in Fig. 3 and show that the rescued RNPs have a cap-snatching defective phenotype.

The rescue of viral RNPs containing the mutant R142A polymerase complex, as described above, enabled us to purify these RNPs and use them as templates for a second in vivo reconstitution experiment in which instead of a template RNA we introduced the rescued and purified R142A mutant RNPs in the system. This strategy ensured that only replication-defective RNPs are used as templates for in vivo replication and allowed us to ask whether the resident polymerase complex or a distinct, soluble polymerase is responsible for replication of RNPs in vivo. The concentration and biological activity of these purified RNPs was first controlled by Western-blot and in vitro transcription. The results are presented in Fig. 3B and show that higher yields were obtained for RNPs containing the E361A mutation in PB2 than those containing the R142A mutation. This was expected, as the latter could only be amplified by trans-complementation (see Fig. 1 above). The transcription phenotype of these purified RNPs was in agreement with the mutations present in PB2 (Fig. 3B, right panel). Therefore, cultures of HEK293T cells were co-transfected with purified RNPs containing either the R142A mutation or the E361A mutation in PB2, plasmids encoding PB1, PA, NP and a plasmid encoding either PB2-His R142A (replication-defective) or PB2-His E361A (transcription-defective) (see Fig. 3A for a diagram of the experimental setting). As controls, the RNPs were co-transfected with empty pCMV vector or the expression plasmids were transfected in the absence of template RNPs. The intracellular accumulation of progeny RNPs was determined by Ni^2+^-NTA-agarose purification, Western-blot and in vitro transcription as indicated above and the results are presented in Fig. 3C and Fig. 4. The cultures co-transfected with RNPs E361A and plasmids including PB2 E361A (Fig. 3C; RNP361-Pol361) served as positive control and, indeed gave rise to the accumulation of RNPs to levels similar to the standard, wt system (see Fig. 1B, HisPB2). No background was observed when template RNPs were transfected (Fig. 3C; RNP142/CMV, RNP361/CMV). A fraction of the NP expressed was retained in the Ni^2+^-NTA-agarose resin (Fig. 3C; Pol142, Pol361) and defined the background level of the purification protocol (but see Fig. 4 below). The co-transfection of RNPs containing mutation PB2 R142A and the same mutant plasmids yielded no increase above background in the level of purified RNPs (Fig. 3C; RNP142/Pol142) but the mixed transfection of RNPs with the mutation PB2 R142A and the expression plasmids with mutation PB2 E361A led to a high level of replication (around 80% of control values) (Fig. 3C; RNP142/Pol361). To verify these results and to determine the polarity of the progeny RNA, similar experiments were carried out and the RNA present in the purified his-RNPs was analysed by hybridisation with positive- and negative-polarity RNA probes comprising the NS sequence. The results reinforced the data obtained by Western-blot and indicated that most of the progeny RNPs are vRNPs (Fig. 4), as previously reported [15]. The accumulation and phenotype of the progeny RNPs was also verified by in vitro transcription using either ApG or β-globin as primers (Fig. 5). The accumulations observed paralleled those shown in Fig. 3 but the background levels from samples Pol142, Pol361 and RNP142-Pol142 were negligible. Much higher activity levels were obtained with ApG primer, indicating that the progeny RNPs contained PB2 with mutation E361A. The results presented in Figs. 3 and 4 indicate that a polymerase complex distinct from that present in the template RNP can perform the replicative synthesis of viral RNA. The high level of replication detected by trans-complementation suggests that virus RNA replication mostly occurs in trans. It could be argued that the mutation R142A in PB2 might destabilise the polymerase-promoter complex, allowing the efficient replacement by a polymerase complex containing the E361A mutation. However, RNPs containing the R142A mutation are as efficient in transcription as wt RNPs, suggesting that they are not affected in promoter recognition.

### Transcription of vRNPs takes place *in cis* and cannot be stimulated by non-resident polymerase complexes *in trans*

**Figure 6:**
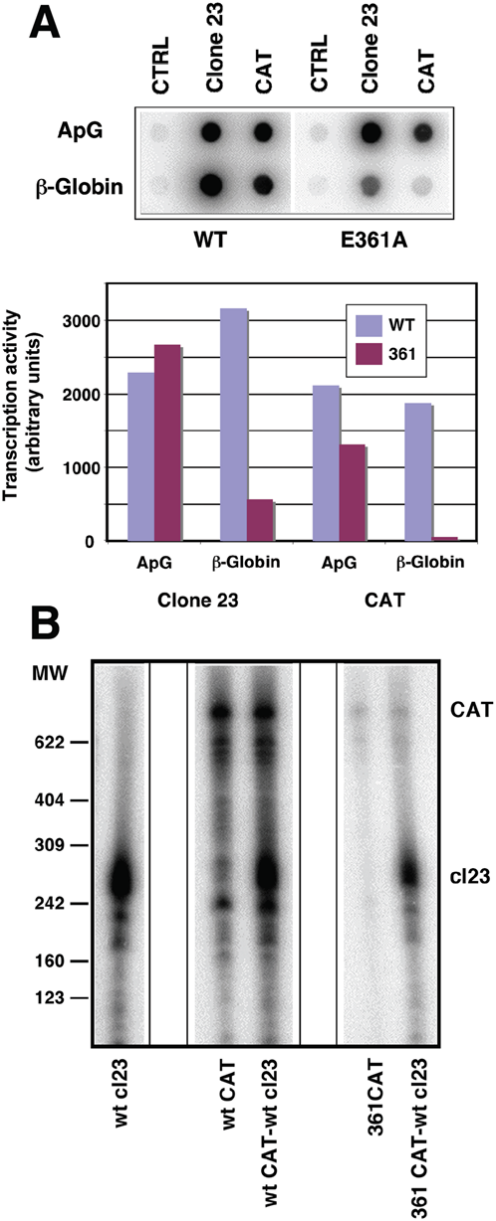
Genetically distinct RNPs cannot transcribe reciprocally in vitro. (A) Recombinant RNPs were generated by in vivo amplification as described in Materials and Methods and the legend to Fig. 1, using either wt (WT) or transcription-defective (E361A) polymerase. Short (clone 23 -248 nt-), long (CAT -720 nt-) or no (CTRL) RNA replicons were used. The RNPs were purified by Ni^2+^-NTA-agarose chromatography and their transcription activity was determined. Top panel shows the phosphorimager data and bottom panel presents the quantisation, indicating the cap-snatching defective phenotype of RNPs containing the E361A mutation in PB2. (B) Purified wt (WT CAT) or E361A mutant RNPs (361 CAT) containing the *cat* gene were transcribed in vitro, using β-globin mRNA as primer, either alone or in a mixture with wt clone 23 RNPs (cl23). The transcription products were purified and analysed by polyacrylamide-urea denaturing gel electrophoresis. The mobility of molecular weight markers is shown to the left and the position of *cat* and clone 23 transcripts is indicated to the right.

It is well established that vRNPs can transcribe mRNAs in the absence of any newly synthesised viral proteins (primary transcription) [39],[40] and highly purified recombinant RNPs can transcribe in vitro [18] (R. Coloma, unpublished results). However, it is not clear whether transcription takes place intramolecularly, i.e. in cis, or a RNP can transcribe another RNP. To test this possibility we reconstituted in vivo two genetically distinct RNPs, one containing a *cat* virus replicon (with the *cat* negative-polarity ORF flanked by the UTRs of the NS segment of influenza virus), the other one being the NS deletion mutant clone 23 [14],[15]. Both RNPs contained a His-tagged PB2 subunit to allow purification by affinity chromatography as indicated above but two PB2 alleles were used, either wt or mutant E361A, which is defective in the recognition of the cap structure [20]. Purified RNPs were used either separately or in combination for in vitro transcription with ApG or β-globin as primers and the transcription products were analysed by denaturing polyacrylamide gel electrophoresis and autoradiography. The results are presented in Fig. 6. As expected, the purified wt RNPs were active, both when ApG or β-globin were used as primers (Fig. 6A). The RNPs containing the mutation PB2 E361A could transcribe mRNA with ApG as primer, but did so less efficiently when using β-globin mRNA as primer donor (Fig. 6A). These results allowed us to test whether a purified, wt clone 23 RNP could rescue the transcription activity of mutant E361A *cat* RNP *in trans*, since the mRNA products would be distinguishable by size (720 nt versus 248 nt). The wild-type *cat* RNPs could transcribe efficiently, both when incubated on their own and when mixed with clone 23 RNPs (Fig. 6B, middle panel). The *cat* RNPs containing PB2 E361A only produced background transcription levels and no increase in the amount of *cat* mRNA was observed when wt clone 23 RNP was co-transcribed (Fig. 6B, right panel). Quantisation of the relevant bands indicated that the increase in *cat* transcript in the co-transcription of clone 23 RNP+E361 *cat* RNP versus the transcription of E361 *cat* RNP was less than 3% of the *cat* transcript value obtained by co-transcription of clone 23 RNP+wt *cat* RNP. These results suggest that, at least in vitro, no transcription *in trans* among different RNPs takes place.

**Figure 7:**
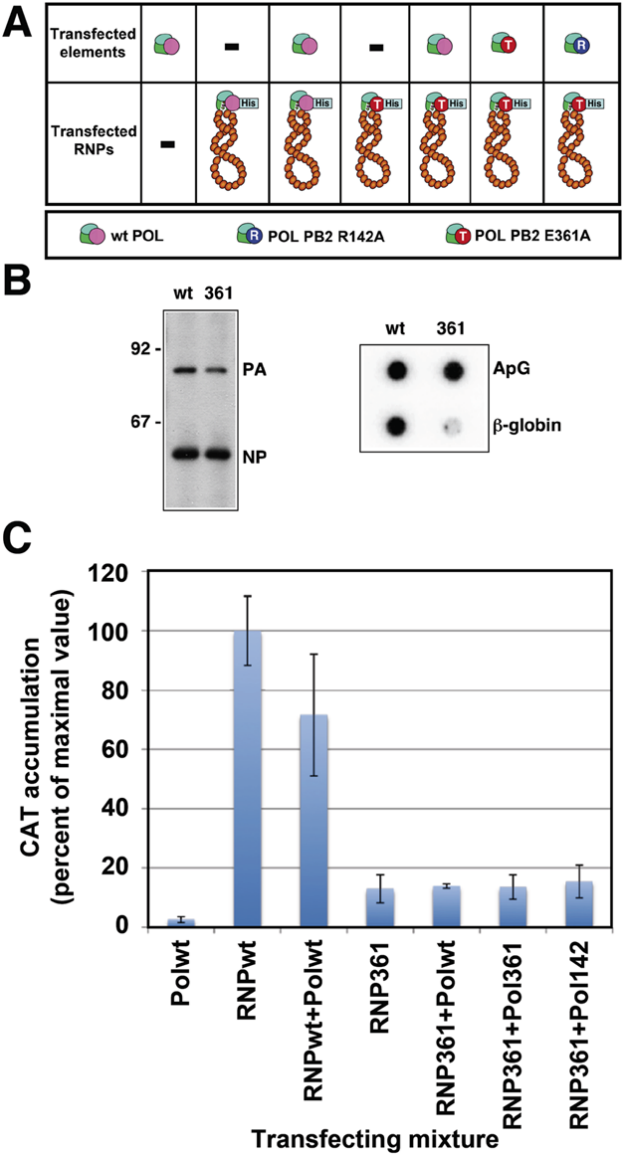
Lack of transcription of RNPs by a soluble polymerase complex *in trans*. (A) Cultures of HEK293T cells were transfected with plasmids expressing various combinations of the polymerase subunits as indicated in the diagram. In some cases, purified *cat* RNPs containing wt or transcription-deficient polymerase were also transfected. (B) Left panel. The amount of wt (wt) or transcription deficient (361) RNPs transfected was ascertained by Western-blot with anti-NP and anti-PA antibodies. The mobility of molecular weight markers is shown to the left and the position of PA and NP proteins is indicated to the right. Right panel. The transcription phenotype of the RNPs transfected was determined by in vitro transcription using ApG (ApG) or β-globin mRNA (β-globin) as primers. The panel shows the phosphorimager data. (C) The amount of CAT protein accumulated in the cells was determined by ELISA. The data are presented as percent of the value obtained by transfection of purified wt RNPs and represent the average and standard deviation of six independent experiments. The 100% value represented corresponds to a concentration of 340 pg/ml of CAT protein.

However, the possibility still persists that a soluble polymerase complex is able to transcribe a vRNP template *in trans*. To analyse this alternative we generated in vivo recombinant RNPs containing the negative polarity *cat* virus replicon, purified them by affinity chromatography as indicated above and used them to transfect HEK293T cultures. Alternatively, the cultures were co transfected with the purified *cat*-containing RNPs and plasmids expressing the polymerase subunits (see Fig. 7A for a diagram of the experiment). As no plasmid expressing NP was used, no in vivo replication of the RNPs can take place [41],[42]. Two RNP versions were used, either wt or transcription-defective (containing PB2 E361A mutant). Three alternative alleles were used to express in vivo PB2, generating wt polymerase, transcription-defective E361A or replication-defective R142A polymerase complexes, and various RNP-polymerase combinations were used in co-transfection experiments. In this way the experiment would mimic the situation of primary transcription (transfection of purified RNPs) or secondary transcription (co-transfection of RNPs with plasmids expressing the polymerase complex). At 24 hours post-transfection total cell extracts were prepared and the CAT protein accumulation was determined by ELISA. To ensure that the purified RNPs used for transfection were biologically active, two assays were carried out. First, their transcription activity was determined in vitro. As shown in Fig. 7B, there was a good correlation between the concentration of the RNPs, as determined by Western-blot with anti-NP and anti-PA antibodies, and the their capacity to synthesise RNA in vitro. Furthermore, the relative activity when using ApG or β-globin mRNA as primers verified that the purified mutant RNPs contained the E361A mutation (Fig. 7B, 361). In addition, the biological activity of the purified 361 RNPs was verified in vivo, by their co-transfection with plasmids expressing the polymerase subunits and NP. The results are presented in Fig. S4 and indicate that they can serve as templates for replication and transcription in vivo. Expression of the polymerase subunits did not yield any detectable CAT protein, as expected (Fig. 7C, Pol wt), but the transfection of wt purified RNPs lead to clearly measurable CAT accumulation (Fig. 7C, RNP wt) and co-expression of wt RNPs with wt polymerase did not lead to any increase of CAT accumulation (Fig. 7C, Pol wt-RNP wt). As control transfections with CAT-containing cellular extracts indicated that the carry-over of the protein was in the range of 10^−3^ to 10^−4^ (data not shown), the CAT protein generated by transfection of wt purified RNPs should represent primary transcription. In agreement with their transcription-defective phenotype, transfection of purified mutant 361 RNPs produced much less CAT accumulation (Fig. 7C, RNP 361). No significant increase in the level of CAT protein was observed by co-transfection of the RNPs containing the E361A mutation with polymerase-expressing plasmids, neither wt nor mutant polymerase and no correlation was observed between the accumulation of CAT and the phenotype of the polymerase co-expressed (Fig. 7C; compare Pol wt vs Pol 142 vs Pol 361+RNP 361). These results indicated that, under the experimental conditions used, no *trans*-activation of transcription occurs *in vivo*.

## Discussion

**Figure 8:**
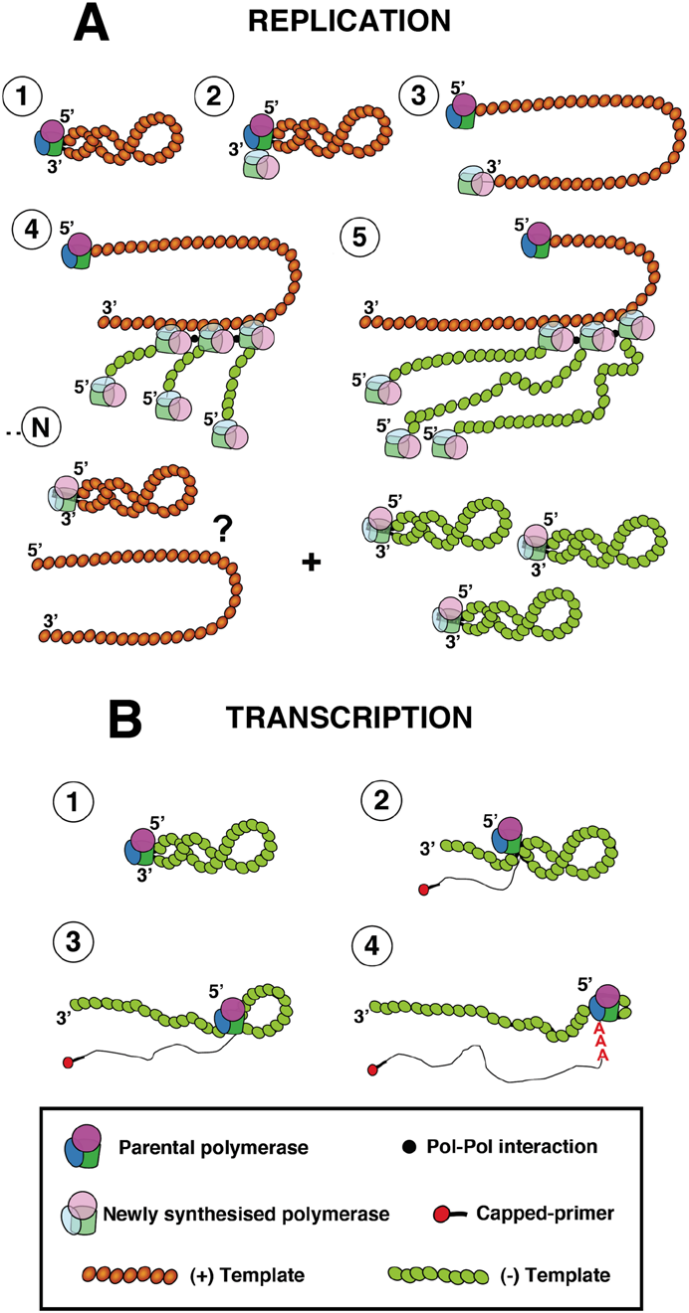
A model for influenza RNP replication and transcription. (A) Various steps in the process of RNP replication. The coloured NP indicates the polarity of the templates (brown: positive polarity; green: negative polarity). The parental polymerase complex is denoted by solid colours while the semi-transparent colouring indicates a newly synthesised complex. See text for details. (B) Various steps in the process of RNP transcription. The capped primer is depicted as a thick line with a red circle. See text for details.

The processes of virus RNA replication and transcription usually require the action of one to several virus-specific proteins, notably the RNA-dependent RNA polymerase (RdRp), and various host cell factors (for a review see [43]. To unravel the complex procedures involved, genetic experimental approaches have been particularly useful. For example, genetic data have strongly supported the requirement of RdRp oligomerisation for RNA replication in several virus groups, like poliovirus [44],[45], HCV [46],[47] and Sendai virus [48],[49]. Early studies on the dominance of RNA-synthesis negative ts mutants of VSV suggested that the oligomerisation of virus factors involved in RNA replication is an essential step in the process [50], a conclusion that could be also verified in the poliovirus system [51]. More generally, the multimeric nature of complex viral systems, as the virus particles, has profound consequences in the apparent phenotype observed [52],[53]. In the case of influenza, early data on the intragenic complementation of mutants affecting the PB1 and PA proteins suggested the potential role of virus polymerase interactions in the infectious cycle [54]–[56] and the recent biochemical evidence for virus polymerase oligomerisation supported such contention [57]. Here we have taken advantage of the availability of well-established recombinant systems for RNP replication and transcription and well-characterised polymerase mutants to address specific questions on the mechanisms of these processes. Due to the segmented nature of the influenza virus genome it is essential to use mutant polymerases having phenotypically distinct mutations in the same subunit, thus avoiding the problems of reassortment. Hence, we have used point mutants of polymerase PB2 subunit that abolish RNA replication but transcribe normally (R142A or F130A) [36] and/or mutants that are defective in cap-recognition and transcribe poorly, but replicate virus RNA normally (E361A among others) [20]. With these experimental tools we have asked whether the polymerase complex present in an RNP actually perform the replicative or transcriptional synthesis of RNA and whether the polymerase complex present in the progeny RNP is identical to that performing replicative synthesis of RNA. Our results will be discussed on the basis of the model presented in Fig. 8, in which only the replication step cRNP-to-vRNP is presented. The results shown in Figs. 1 and 2 indicated that two such phenotypically distinct mutant polymerases can complement to perform viral RNP replication in vivo and demonstrated that a replication-defective polymerase can be incorporated into progeny RNPs. These results are consistent with the model presented in Fig. 8A, step 4, that suggest that a polymerase complex distinct from that performing replicative synthesis is involved in the recognition of the 5′-end of the progeny vRNA. This model is also consistent with the results published earlier indicating that a pre-expressed polymerase can protect newly synthesised cRNA [13],[38]. The identity of the replicative polymerase complex could be tested by directly transfecting mutant RNPs as templates for the replication reaction and asking whether co-expressed replication-defective or transcription-defective polymerase complexes could carry out the replication process in trans. The results shown in Figs. 3 and 4 demonstrated that a polymerase complex genetically distinguishable from that present in the parental RNP was able to perform replication and became incorporated into the progeny RNPs. These results are compatible with the model presented in Fig. 8A, steps 2–4, whereby a soluble polymerase complex would interact with that resident in the parental RNP and gain access to the 3′-terminal sequence in the promoter. Such polymerase-polymerase interaction is supported by the genetic data presented here, by the intragenic complementation reported earlier [54],[55] and by the oligomerisation of influenza polymerase in vivo [57]. Although not shown in Fig. 8A, we can not exclude that a host factor(s) participate in the polymerase-polymerase interaction and in fact several nuclear factors have been described previously that could play such a role [25]–[30],[32]. The trans-replication model depicted in Fig. 8A, steps 2–4 relates to the cRNP-to-vRNP phase in replication. However, earlier data published on the protection of newly synthesised cRNA by pre-expressed polymerase would suggest that the vRNP-to-cRNA phase can occur in cis, since a pre-expressed, catalytically inactive polymerase allowed the accumulation of cRNA in cicloheximide-treated, virus-infected cells [13].

According to the model proposed here, the soluble polymerase complex would act as replicative enzyme by de novo initiation and elongation through the NP-RNA template (Fig. 8, steps 3–4). We propose that the 3′-end of the parental RNA is used repetitively for further initiation rounds, thereby leading to several progeny vRNPs generated from a single cRNP template. For simplicity, the model presented in Fig. 8A does not show the interaction of the new incoming replicative complex with the parental polymerase bound to the 5′-end of the template, but such interaction might be required. In view of our previous evidence on polymerase-polymerase interaction [57], an appealing possibility is that the replicative polymerase complexes would oligomerise to form a fixed replication platform along which the NP-RNA template would move 3′-to-5′ to generate many progeny vRNPs. Such strategy has a precedent in other positive-strand RNA viruses [44]–[47] and would be consistent with the localised synthesis of influenza virus RNA in the nucleus [58],[59]. A critical point in the generation of progeny vRNP is the recognition and packaging of its newly synthesised 5′-end. Our results are compatible with the proposal that a polymerase complex distinct from the replicative enzyme can protect the newly synthesised vRNA (Fig. 8A, step 4) and this event probably represents the sequence-specific step in the encapsidation of RNA into progeny RNP. The subsequent incorporation of successive NP monomers would be directed by polymerase-NP interactions [60], that have been shown as essential for RNP replication [61],[62], as well as by the NP-NP oligomerisation [17] (R. Coloma, unpublished results). Another critical point in the replication process is the displacement of the parental polymerase complex bound to the 5′-end of the template, a step necessary to avoid polyadenylation (see below) and to allow the complete copy of the RNA. Our results do not permit us to distinguish whether the elimination of such interaction is transient or permanent, but an attractive possibility would be that the reiterative copy of the NP-RNA template on a fixed platform of replicative polymerases would force the displacement of the parental polymerase bound to the 5′-terminal sequence. Such displacement could be transient, leading to the replacement of the parental polymerase by each successive replicative polymerase, or permanent, leading to a linearised NP-RNA complex (Fig. 8A, step n).

In contrast to the positive complementation obtained for the replication process, no trans-complementation could be detected in the transcription assays using either in vitro (Fig. 6) or in vivo experiments (Fig. 7). In vitro transcription of a recombinant RNP containing a cap-binding defective polymerase could not be rescued by a wt RNP holding a template of different length (Fig. 6). Similar negative results were obtained in vivo, by transfection of a cap-binding defective RNP and co-expression of wt or replication-defective but transcriptionally functional polymerase (Fig. 7). These results are not compatible with the possibility of transcription among viral RNPs in trans and do not support the possibility of a soluble polymerase transcribing an independent RNP. Furthermore, these results indicate a high stability of the polymerase binding to the RNP structure during the transcription process, as no polymerase exchange could be functionally detected. In view of the lack of detectable transcription in trans, we propose the model presented in Fig. 8B for the generation of viral mRNAs. The resident polymerase complex would be transcriptionally activated by recognition of the cap-containing cellular mRNA and proceed to cap-snatching and elongation of the virus transcript (Fig. 8B, step 2), but still keeping hold of the 5′-terminal sequence of the promoter [63]. Such process would lead to a running knot structure with a diminishing loop length (Fig. 8B, steps 2–4) until the transcribing polymerase reaches the oligo-U polyadenylation signal [10]. Due to steric hindrance, the polymerase would stutter around the oligo-U sequence and generate a 3′-terminal polyA (Fig. 8B, step 4). For simplicity, the model presented in Fig. 8B shows the transcribing RNP in a linearised form, but the polymerase complex should recognise the 3′-terminal side of the promoter at some time later in the process, in order to recycle and allow further rounds of transcription. This model for transcription of RNPs in cis is compatible with the fact that parental RNPs perform primary transcription as a first step in the infection and with the possibility to rescue virus by transfection of purified virion and/or recombinant RNPs [64],[65]. It also would fit with the correlation of vRNA and mRNA levels of the various RNA segments along the infection cycle [66],[67].

In summary, using genetically distinct RNA polymerase complexes, we have presented direct evidence for trans-complementation during the influenza virus RNA replication process. These results are compatible with a new model for viral RNA replication whereby a template RNP would be replicated in trans by a soluble polymerase complex and the progeny RNP encapsidation would be specified in trans by a polymerase complex distinct from the replicative enzyme. In contrast, no transcription in trans could be detected in vitro or in vivo and hence we propose a model for cis-transcription of the RNPs whereby the resident polymerase complex would be responsible for mRNA synthesis and polyadenylation.

## Materials and Methods

### Biological materials

The HEK293T cell line [68] was used throughout. The origins of plasmids pCMVPB1, pCMVPB2, pCMVPB2His, pCMVPA and pCMVNP, as well as pHHclone 23, have been described [20],[64]. Plasmid pHHCAT was kindly provided by A. Rodriguez. The antibodies specific for PB2 and PA have been described [69],[70]. Antibodies specific for NP were prepared by immunisation with purified His-NP protein. Mutant PB2 plasmids including mutations in the N-terminal region [36] or the cap-binding site [20] have been reported earlier. The mutations F130A, R142A, E361A, H357A, K370A and F404A were transferred to pCMVPB2 by swapping the appropriate restriction fragments. The genotype of the mutant plasmids was verified by sequencing.

### Amplification and purification of recombinant RNPs

Recombinant RNPs containing either the ΔNS clone 23 (248 nt) or the NSCAT (720 nt) genomic RNAs were generated and amplified in vivo by transfection of plasmids pCMVPB1, pCMVPB2His, pCMVPA, pCMVNP and either pHHclone23 or pHHNSCAT into HEK293T cells, using the calcium phosphate protocol [71]. For RNP purification, cell extracts were prepared at 24 hours post-transfection and incubated overnight at 4°C with 30 µl of Ni^2+^-NTA-agarose resin in a buffer containing 50 mM Tris-HCl-100 mM KCl-5 mM MgCl2-0.5% Igepal-20 mM imidazol-1 u/µl RNAsin-EDTA-free protease inhibitors cocktail, pH 8. The resin was washed with 100 volumes of 50 mM Tris-HCl-100 mM KCl-5 mM MgCl2-0.5% Igepal-20 mM imidazol, pH 8 and eluted with 50 mM Tris-HCl-100 mM KCl-0.5% Igepal-175 mM imidazol, pH 8. Under these conditions, binding of the progeny RNPs to the resin was quantitative, as using three-fold excess of Ni^2+^-NTA-agarose did not result in any increase in the yield of purified RNPs (see Fig. S5).

### Biochemical techniques

Western-blotting was performed as described [30]. The replication of RNPs in vivo was determined as described [20]. In brief, cultures of HEK293T cells were transfected with plasmids pCMVPB1, pCMVPB2His (or mutants thereof) or pCMVPB2 (or mutants thereof), pCMVPA, pCMVNP and pHHclone 23. In some experiments pHHclone 23 plasmid was omitted and purified clone 23 RNPs were transfected instead, 24 hours after plasmid transfection. Total cell extracts were prepared at 24 hours post-transfection and used for purification by affinity chromatography on Ni^2+^-NTA-agarose as indicated above and the accumulation of progeny RNPs was determined by Western-blot with anti-NP-specific antibodies. The transcription of RNPs in vivo was assayed by transfection of purified NSCAT RNPs into HEK293T cells. The cultures were first transfected with plasmids pCMVPB1, pCMVPB2 (or mutants thereof) and pCMVPA [20] and 24 hours later were further transfected with purified His-tagged NSCAT RNPs under the same conditions. At 24 hours post RNP-transfection, total cell extracts were prepared and the CAT protein concentration was determined by ELISA (GE Healthcare).

### RNA analyses

To determine the transcription activity of purified RNPs, samples were incubated in a buffer containing 50 mM Tris-HCl-5 mM MgCl2-100 mM KCl-1 mM DTT-10 µg/ml actinomycin D-1 u/µl RNAsin-1 mM ATP-1 mM CTP-1 mM UTP-10 µM α-P^32^-GTP (20 µCi/µmol) and either 100 µM ApG or 10 µg/ml β-globin mRNA, for 60 min at 30°C. The RNA synthesised was TCA precipitated, filtered through a nylon filter in a dot-blot apparatus and quantified in a phosphorimager. To analyse the transcription products, similar reactions were carried out but the specific activity of the labelled GTP was increased to 200 µCi/µmol. The synthesised RNA was isolated by treatment with proteinase K (50 µg/ml) for 30 min at 37°C in TNE-1% SdS and phenol extraction. The RNA was ethanol precipitated, resuspended in formamide loading buffer and analysed by electrophoresis in a 4% polyacrylamide-urea denaturing gel.

To analyse the progeny RNA, purified RNPs were incubated with proteinase K (200 µg/ml) in a buffer containing 100 mM NaCl-5 mM EDTA-0.5% SDS-50 mM Tris.HCl, pH 7.5 for 60 min at 37°C, phenol extracted with ethanol precipitated. Samples of the purified RNAs were denatured by boiling for 3 min in 7.5% formaldehyde-10SSC and were fixed onto nylon filters. Replicate filters were hybridised at 37°C with full-length NS riboprobes of either positive- or negative-polarity in a buffer containing 6SSC-40% formamide-0.5% SDS-5xDenhart's mixture-100 µg/ml single-stranded DNA. After washing at 60°C with 0.1SSC-0.1%SDS, hybridisation signals were quantitated in a phosphorimager.

## Supporting Information

Figure S1 Expression of wild-type and mutant PB2 proteins. Cultures of HEK293T cells were transfected with plasmids encoding wt or mutant PB2 proteins, as indicated. Total cell extracts were prepared and analysed by Western-blot using anti-PB2 antibodies as described in Materials and Methods. The position of the PB2-specific signals of His-tagged (PB2His) or untagged PB2 is indicated to the right.
(0.30 MB TIF)

Figure S2 Intracistronic polymerase complementation during influenza virus RNA replication. Cultures of HEK293T cells were transfected with plasmids expressing a virus-like replicon of 248 nt, the NP and various combinations of the polymerase subunits as indicated (replication-defective -R142A, F130A-; transcription-defective -H357A, K370A, F404A-). The progeny RNPs were purified from total cell extracts over Ni-NTA-agarose resin and analysed by Western-blot with anti-NP antibodies. The top panel presents the accumulation of NP in the total cell extract whereas the bottom panel shows the NP accumulation of purified RNPs. In the bottom graph the quantitation of the data is presented as percent of maximal value.
(0.59 MB TIF)

Figure S3 Phenotype of trans-complemented RNPs. The purified RNP preparations presented in Fig. S2 were tested for in vitro transcription primed with either ApG (red) or β-globin mRNA (green). The data are presented as percent of maximal value. The transcription activities parallel the values of NP accumulation presented in Fig. S2 and show that the rescued RNPs have a wt cap-snatching phenotype.
(0.51 MB TIF)

Figure S4 Control of the biological activity of transfected RNPs. To verify the biological activity of the PB2 E361A RNPs used in the experiments described in Fig. 7, cells were transfected with polymerase subunits and NP-expressing plasmids and further transfected with the purified RNPs. At 24 h post-transfection of the latter cell extracts were prepared and the CAT protein accumulation was determined by ELISA. As control, single transfection with polymerase subunit and NP-expressing plasmids was performed in parallel.
(0.96 MB TIF)

Figure S5 Linearity of the RNP binding to Ni^2+^-NTA-agarose resin. Cultures of HEK293T cells were transfected with plasmids expressing PB1, PB2, PA, NP and a model vRNA template (ΔNS clone 23). At 24 h post-transfection, cell extracts were prepared and used for affinity chromatography over Ni^2+^-NTA-agarose resin as described under Materials and Methods. Aliquots of the input extract (IN), material not bound to the resin (NB) and eluted with imidazol (EL), were analysed by Western-blot with antibodies specific for PA and NP. Increasing amounts of resin, 30, 60, and 100 µl were used for identical input extracts, as indicated at the top of the Figure. The mobility of molecular weight markers (in kDa) is shown to the left. The position of the signals specific to PA and NP are indicated to the right.
(0.59 MB TIF)
