# Supplementary figures and images for: Genetic trans-Complementation Establishes a New Model for Influenza Virus RNA Transcription and Replication
Source: PLoS Pathog. 2009 May 29;5(5):e1000462. doi: 10.1371/journal.ppat.1000462 (PMC2682650; doi:10.1371/journal.ppat.1000462)

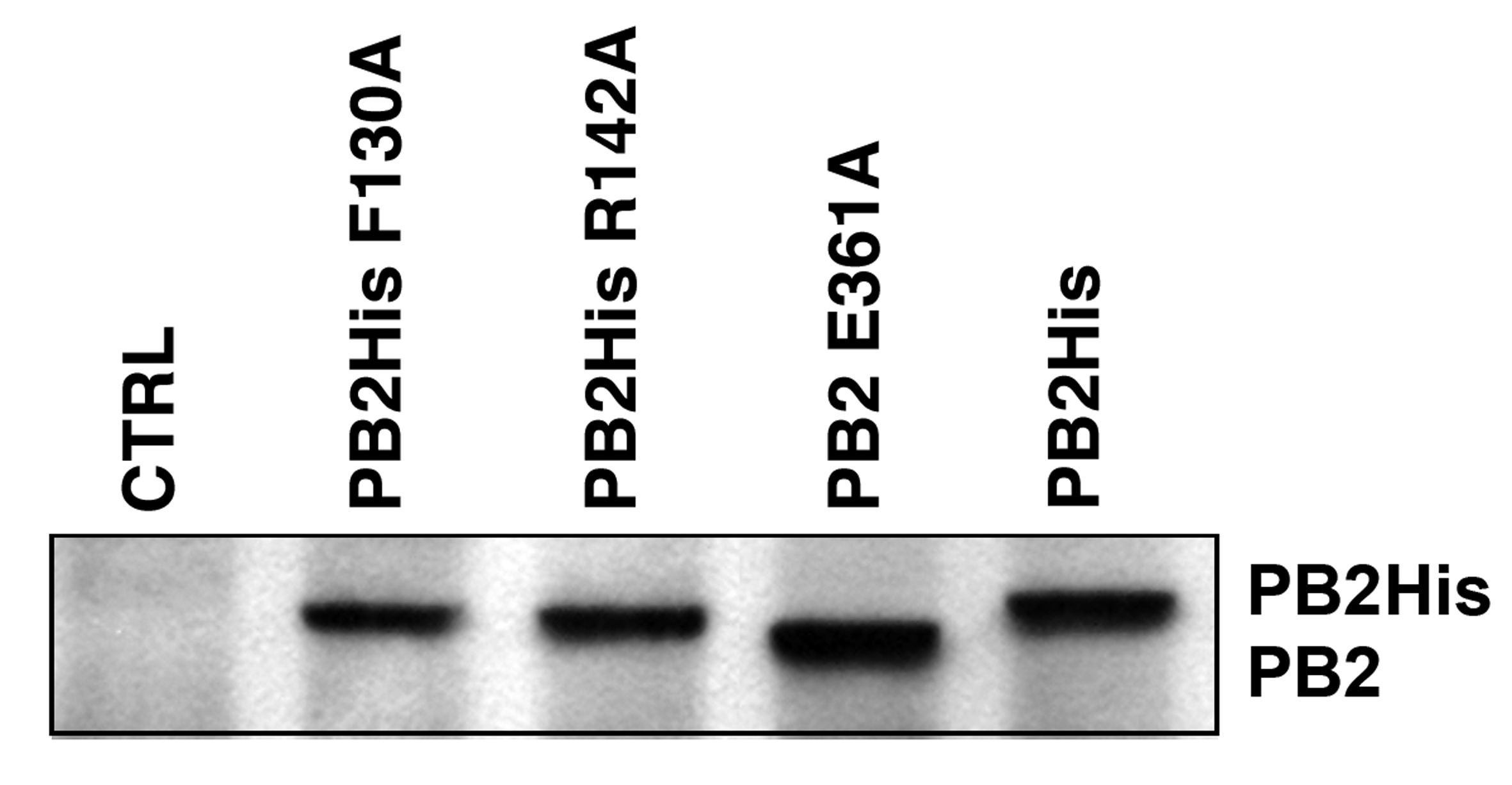

Supplement: Figure S1 — Expression of wild-type and mutant PB2 proteins. Cultures of HEK293T cells were transfected with plasmids encoding wt or mutant PB2 proteins, as indicated. Total cell extracts were prepared and analysed by Western-blot using anti-PB2 antibodies as described in Materials and Methods. The position of the PB2-specific signals of His-tagged (PB2His) or untagged PB2 is indicated to the right. (0.30 MB TIF) [file ppat.1000462.s001.tif]

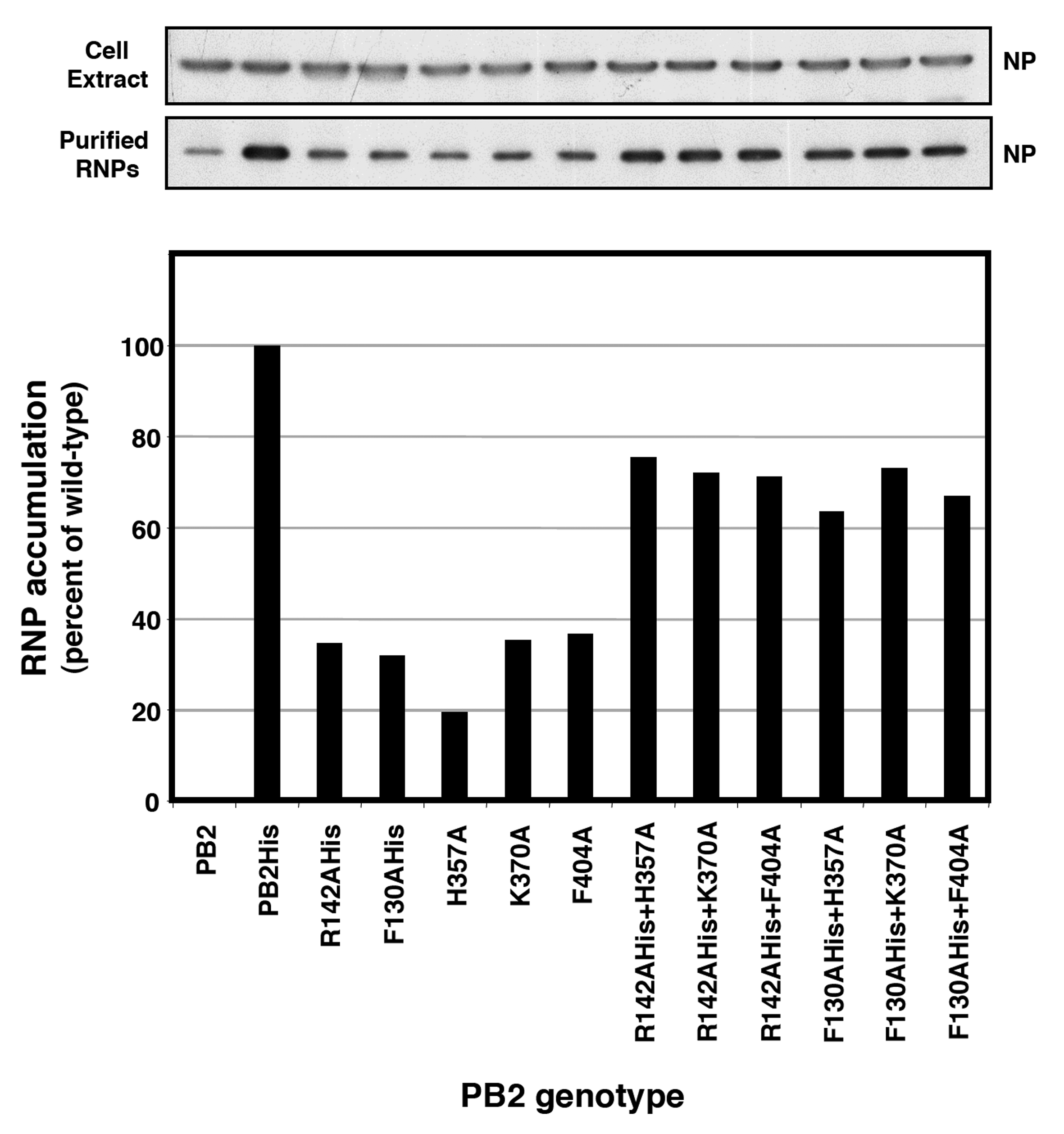

Supplement: Figure S2 — Intracistronic polymerase complementation during influenza virus RNA replication. Cultures of HEK293T cells were transfected with plasmids expressing a virus-like replicon of 248 nt, the NP and various combinations of the polymerase subunits as indicated (replication-defective -R142A, F130A-; transcription-defective -H357A, K370A, F404A-). The progeny RNPs were purified from total cell extracts over Ni-NTA-agarose resin and analysed by Western-blot with anti-NP antibodies. The top panel presents the accumulation of NP in the total cell extract whereas the bottom panel shows the NP accumulation of purified RNPs. In the bottom graph the quantitation of the data is presented as percent of maximal value. (0.59 MB TIF) [file ppat.1000462.s002.tif]

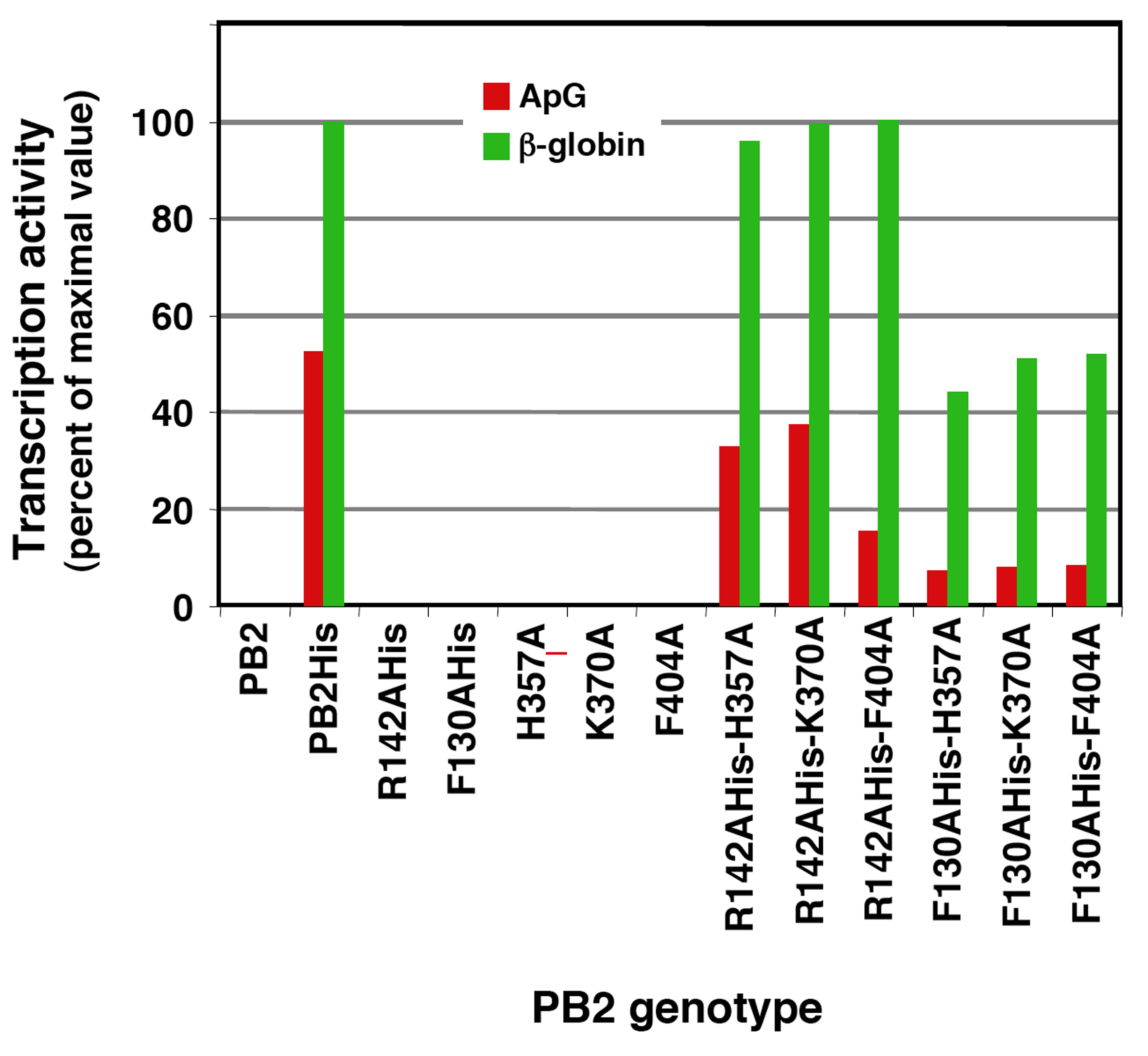

Supplement: Figure S3 — Phenotype of trans-complemented RNPs. The purified RNP preparations presented in Fig. S2 were tested for in vitro transcription primed with either ApG (red) or β-globin mRNA (green). The data are presented as percent of maximal value. The transcription activities parallel the values of NP accumulation presented in Fig. S2 and show that the rescued RNPs have a wt cap-snatching phenotype. (0.51 MB TIF) [file ppat.1000462.s003.tif]

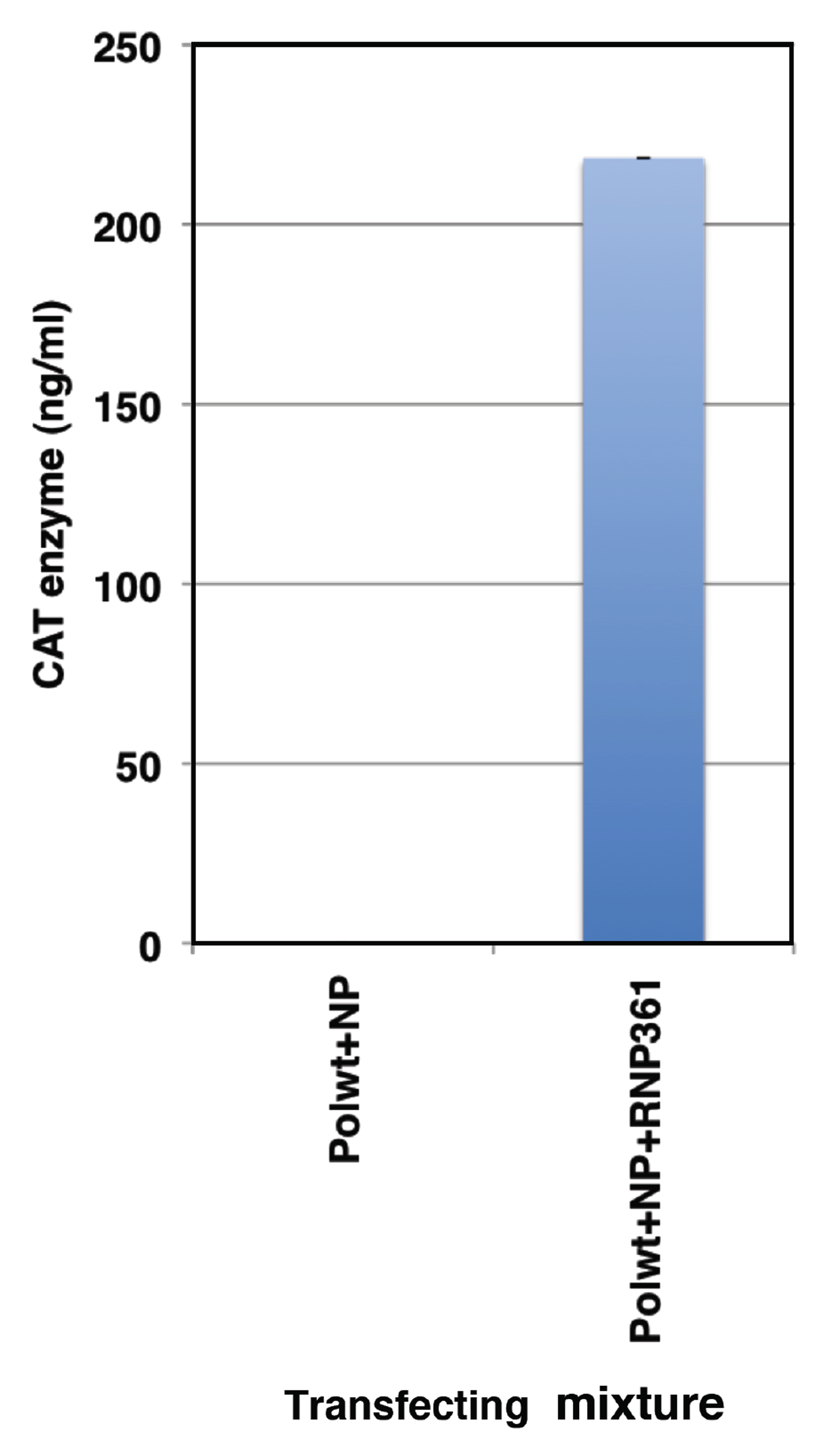

Supplement: Figure S4 — Control of the biological activity of transfected RNPs. To verify the biological activity of the PB2 E361A RNPs used in the experiments described in Fig. 7, cells were transfected with polymerase subunits and NP-expressing plasmids and further transfected with the purified RNPs. At 24 h post-transfection of the latter cell extracts were prepared and the CAT protein accumulation was determined by ELISA. As control, single transfection with polymerase subunit and NP-expressing plasmids was performed in parallel. (0.96 MB TIF) [file ppat.1000462.s004.tif]

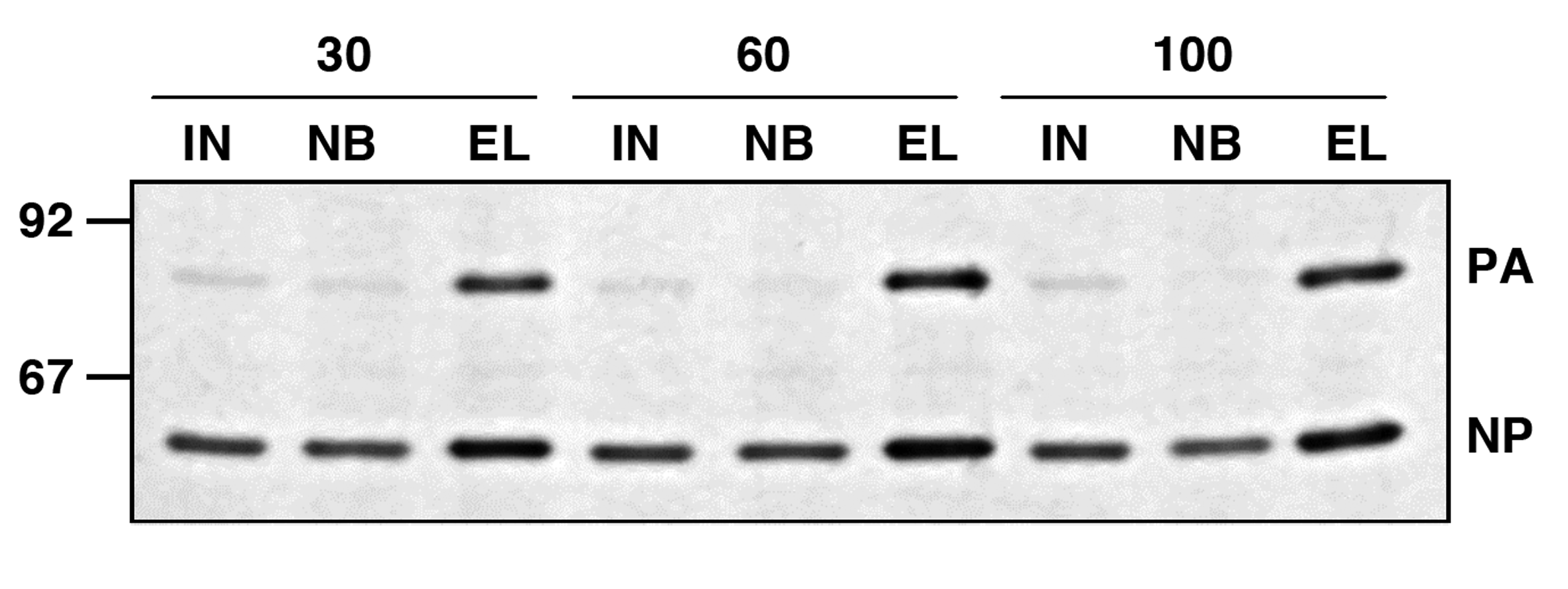

Supplement: Figure S5 — Linearity of the RNP binding to Ni2+-NTA-agarose resin. Cultures of HEK293T cells were transfected with plasmids expressing PB1, PB2, PA, NP and a model vRNA template (ΔNS clone 23). At 24 h post-transfection, cell extracts were prepared and used for affinity chromatography over Ni2+-NTA-agarose resin as described under Materials and Methods. Aliquots of the input extract (IN), material not bound to the resin (NB) and eluted with imidazol (EL), were analysed by Western-blot with antibodies specific for PA and NP. Increasing amounts of resin, 30, 60, and 100 µl were used for identical input extracts, as indicated at the top of the Figure. The mobility of molecular weight markers (in kDa) is shown to the left. The position of the signals specific to PA and NP are indicated to the right. (0.59 MB TIF) [file ppat.1000462.s005.tif]
